# Association of Receipt of Human Papillomavirus Vaccination and Personal History of Cancer Diagnosis in the United States: A Cross-Sectional Study Using NHIS Data

**DOI:** 10.3390/cancers18182945

**Published:** 2026-09-11

**Authors:** Manali Desai, Shreela V. Sharma, Joël Fokom Domgue, Robert Yu, Wenyaw Chan, Charles Darkoh, Sanjay Shete

**Affiliations:** 1 Division of Cancer Prevention and Population Sciences, The University of Texas MD Anderson Cancer Center, Houston, TX 77030, USA; manali.udesai@gmail.com (M.D.); jfokom@mdanderson.org (J.F.D.); 2 Center for Healthy Communities, Department of Epidemiology, The University of Texas Health Science Center at Houston, Houston, TX 77030, USA; shreela.v.sharma@uth.tmc.edu (S.V.S.); charles.darkoh@uth.tmc.edu (C.D.); 3 Department of Biostatistics, The University of Texas MD Anderson Cancer Center, Houston, TX 77030, USA; rkyu@mdanderson.org; 4 Department of Epidemiology, The University of Texas MD Anderson Cancer Center, Houston, TX 77030, USA; 5 Department of Biostatistics and Data Science, The University of Texas Health Science Center at Houston, Houston, TX 77030, USA; wenyaw.chan@uth.tmc.edu

**Keywords:** human papillomavirus vaccine, cancer survivors, human papillomavirus-associated cancers, HPV vaccine, survivorship, care coordination, transition-in-care

## Abstract

**Background**: Cancer survivors are at increased risk for developing subsequent cancers, including HPV-associated cancers, compared to the general population. However, evidence regarding the receipt of HPV vaccination and cancer diagnosis status is limited. **Methods**: We analyzed 2019 and 2022 National Health Interview Survey data, focusing on adults aged 18–45 years who were eligible for HPV vaccination. We performed descriptive statistics and evaluated associations using survey-weighted bivariable and multivariable logistic regression to assess the association between a personal history of any cancer diagnosis and receipt of HPV vaccination, adjusting for sociodemographic and behavioral factors. **Results**: Among 20,917 participants (mean [SD] age (years): 31.41 [13.58]), 489 (2.0%) were cancer survivors; 99 (19.3%) received HPV vaccination. Among 20,423 adults without a cancer diagnosis, 5317 (27.8%) had received HPV vaccination. Five participants had missing data on cancer history. Cancer survivors had significantly lower odds of HPV vaccination compared to adults without a personal cancer history (Adjusted Odds Ratio [aOR]: 0.67, 95% CI: 0.52–0.87, *p* = 0.003). Living in non-metro areas (aOR: 0.77, 95% CI: 0.66–0.89, *p* < 0.001), smokers [current (aOR: 0.82, 95% CI: 0.71–0.94, *p* = 0.005), and former (aOR: 0.75, 95% CI: 0.67–0.84, *p* < 0.001)], non-US-born (aOR: 0.54, 95% CI: 0.47–0.61, *p* < 0.001), uninsured (aOR: 0.60, 95% CI: 0.52–0.69, *p* < 0.001) had significantly lower odds of receiving HPV vaccination. Those who were 18–26 years (aOR: 5.49, 95% CI: 4.73–6.37, *p* < 0.001), non-Hispanic others (aOR: 1.40, 95% CI: 1.11–1.75, *p* = 0.004), females (aOR: 3.18, 95% CI: 2.84–3.56, *p* < 0.001), higher education [some college/bachelor’s education (aOR: 1.57, 95% CI: 1.41–1.74, *p* < 0.001), and graduate degree/professional scholar (aOR: 1.91, 95% CI: 1.65–2.22, *p* < 0.001)], living in rented housing (aOR: 1.18, 95% CI: 1.08–1.29, *p* < 0.001) had significantly higher odds of receiving HPV vaccination. **Conclusions**: HPV vaccination uptake is low among cancer survivors. Targeted interventions such as strong oncologists’ recommendations, patient reminders, and care coordination between oncology and primary care professionals are needed to increase HPV vaccination uptake in this population.

## Simple Summary

Cancer survivors are at increased risk of subsequent HPV-associated malignancies. Children’s Oncology Group Long-Term Follow-Up Guidelines for Survivors of Childhood, Adolescent, and Young Adult Cancers (COG LTFU Guidelines) recommend that cancer survivors receive a 3-dose HPV vaccine series, starting 6 months after cancer treatment. We used data from the National Health Interview Surveys to study how the personal history of cancer diagnosis is associated with the receipt of HPV vaccination. We found that adults with a personal history of cancer diagnosis had significantly lower odds of receipt of HPV vaccination compared to adults without a personal history of cancer diagnosis. Smokers, non-US-born, uninsured, and living in non-metro areas had significantly lower odds of HPV vaccination. While adults aged 18–26 years, females, non-Hispanic others, highly educated, and living in rented houses had significantly higher odds of HPV vaccination. This study emphasizes the need for targeted evidence-based interventions to improve the receipt of HPV vaccination among cancer survivors.

## 1. Introduction

Human papillomavirus (HPV) is the most common sexually transmitted infection (STI) in the United States, infecting nearly all sexually active individuals at some point in their lives [1,2]. Infection with high-risk HPV types, particularly types 16 and 18, is associated with the development of precancerous changes that can progress to cancers in the cervix, anogenital areas, and oropharynx [1,3]. Among individuals with weakened immune systems, including those receiving immunosuppressive therapy such as cancer treatment, the ability to clear HPV infections is reduced, increasing the risk of persistence and progression to HPV-related cancers [4]. Furthermore, HPV antibodies acquired through natural infection provide only modest protection against subsequent HPV infection with the same HPV strain, suggesting that prior HPV infection may not confer reliable immunity [5].

HPV vaccination is highly effective in preventing more than 90% of HPV-attributable cancers, which account for approximately 33,700 cases per year in the United States [1]. The Centers for Disease Control and Prevention’s Advisory Committee on Immunization Practices (ACIP) recommends routine HPV vaccination at ages 11–12 years, with vaccination initiated as early as 9 years, and catch-up vaccination through age 26 years. A two-dose schedule is recommended when vaccination is initiated before age 15 years, whereas a three-dose schedule is recommended for those initiating vaccination at ages 15–26 years and for individuals aged 9–26 years with immunocompromising conditions including cancer survivors. For adults aged 27–45 years who have not been adequately vaccinated, HPV vaccination may be considered through shared clinical decision-making [6]. Importantly, the Children’s Oncology Group Long-Term Follow-Up Guidelines for Survivors of Childhood, Adolescent, and Young Adult Cancers (COG LTFU Guidelines) recommend that cancer survivors receive a 3-dose HPV vaccine series, starting 6 months after cancer treatment [7].

According to the American Cancer Society, a cancer survivor is an individual who has ever received a cancer diagnosis, irrespective of the course of their disease [8]. While cancer remains a major public health concern, the cancer mortality rate in the United States has been steadily declining through 2023, preventing nearly 4.8 million deaths since 1991 due to a reduction in smoking, early cancer detection, and better treatment options [9]. As of May 2025, an estimated 18.6 million cancer survivors were living in the US, accounting for 5.4% of the population [10]. Additionally, in 2026, about 2,114,850 new cancer cases and 626,140 cancer deaths are projected to occur in the United States [9]. Therefore, cancer survivors are a growing part of the United States population, highlighting the increasing importance of HPV vaccination in survivorship care to address their unique health challenges.

Cancer survivors are at increased risk of developing new HPV-associated cancers following treatment for their primary malignancy [11,12,13,14,15]. A study conducted by Ou and colleagues among 374,408 cancer survivors in the United States found that cancer survivors had a 70% increased risk of developing HPV-associated subsequent malignant neoplasms compared with the general population [13]. Despite this elevated risk, some studies have reported lower HPV vaccination rates among cancer survivors compared to the general population [16,17]. For example, a 2017 study of 982 young cancer survivors between the ages of 9 and 26 in the United States found that 23.8% had initiated HPV vaccination, compared to 40.5% of individuals in the general population [16]. These findings suggest that many cancer survivors remain at risk for subsequent HPV-related cancers if they do not receive HPV vaccination as recommended.

Given that HPV vaccination is safe and effective among cancer survivors [18], vaccinating age-eligible cancer survivors may help reduce the future burden of HPV-associated cancers. Social determinants of health, such as insurance, income, employment, and medical debt, affect health during cancer survivorship in the United States [19,20]. Given that most studies have focused on understanding HPV vaccination rates among cancer survivors up to age 26 years [16,17,21], this study aims to determine (1) the prevalence of the receipt of HPV vaccination among adults aged 18–45 years, including cancer survivors, and (2) to examine the association between receipt of HPV vaccination and personal history of cancer diagnosis, adjusting for sociodemographic and behavioral factors. Since the HPV vaccine is Food and Drug Administration-approved for adults up to age 45 [22], these findings will help inform evidence-based strategies to improve HPV vaccination receipt among eligible adults, including cancer survivors, across the cancer care continuum, preventing the development of new HPV-related cancers.

## 2. Materials and Methods

### 2.1. Study Design and Population

The National Health Interview Survey (NHIS), conducted by the National Center for Health Statistics (NCHS) at the U.S. Centers for Disease Control and Prevention (CDC), monitors the health of the population by collecting and analyzing data on a wide range of health topics [23]. For this cross-sectional study, we utilized combined data from the 2019 and 2022 NHIS. NHIS is a cross-sectional household interview survey of the civilian, noninstitutionalized population residing in the 50 states and the District of Columbia at the time of the interview [24,25]. The total household response rate was 61.1% and 49.6% for the 2019 and 2022 NHIS samples, respectively [24,25]. All human participants in NHIS are protected by the Ethics Review Board (ERB) of the NCHS [26]. All the study participants provided informed consent for study participation. The NHIS de-identified data are publicly available; thus, our study was deemed exempt by the University of Texas MD Anderson Institutional Review Board.

**Figure 1:**
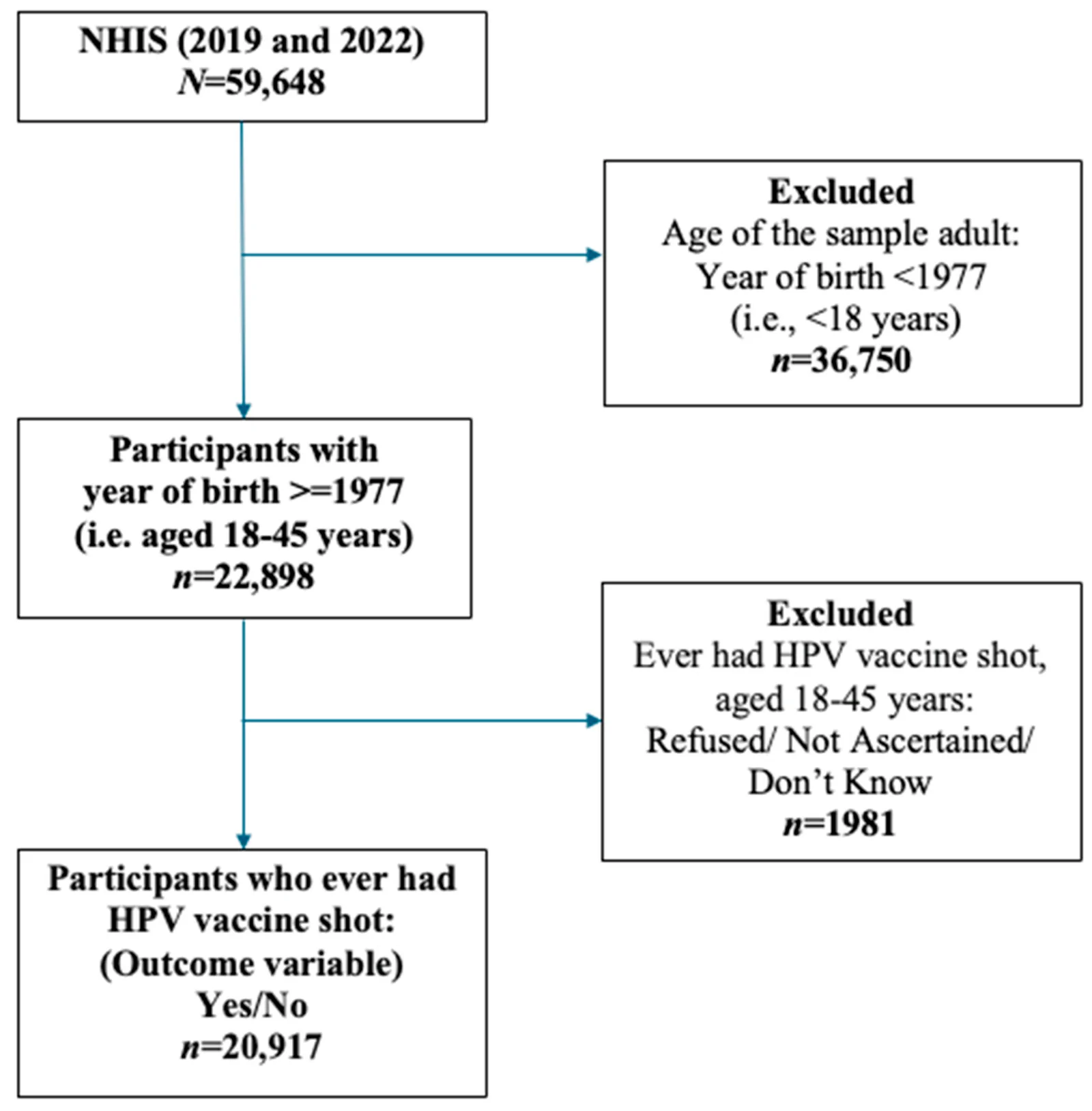
Participant selection flowchart.

The U.S. Centers for Disease Control and Prevention’s Advisory Committee on Immunization Practices (ACIP) recommends routine HPV vaccination beginning at age 11 or 12 years, although vaccination may be initiated as early as age 9 years [27]. Additionally, in 2019, ACIP recommended shared clinical decision-making regarding HPV vaccination for adults aged 27–45 years [28]. Therefore, we restricted our study population to individuals aged 18–45 years who were eligible for HPV vaccination according to ACIP recommendations. A flowchart of the inclusion and exclusion criteria for participants is shown in Figure 1.

### 2.2. Dependent Variable

#### Receipt of HPV Vaccination

The outcome variable was self-reported lifetime HPV vaccination status, assessed by the question: “Have you ever received an HPV shot or vaccine?” Individuals were categorized as 1 if they responded “Yes” and as 0 if they responded “No”. Those who responded “Refused”, “Not Ascertained”, and “Don’t Know” were coded as “missing” and were not included in the analysis [29,30].

### 2.3. Independent Variable

#### Personal History of Any Type of Cancer Diagnoses

The independent variable, self-reported lifetime personal history of any cancer, was assessed by the survey question: “Have you ever been told by a doctor or other health professional that you had cancer or a malignancy of any kind?” We included individuals who responded “Yes” or “No”, whereas those who responded “Refused”, “Not Ascertained”, or “Don’t Know” were coded as “missing” [29,30].

### 2.4. Covariates

Self-reported sociodemographic factors included age (18–26 years, and 27–45 years), sex (male, female), race/ethnicity (non-Hispanic White, non-Hispanic Black, Hispanic, non-Hispanic Asian and non-Hispanic others), educational attainment (up to high school degree, some college/bachelor’s degree including some college without a degree, associate degree and bachelor’s degree, and graduate degree/higher including master’s, professional school degree, or doctoral degree), smoking status (current, former, never), housing status (own, rent), born in US (yes, no), insurance (yes, no), place of residence (metro, combining all large central metro, large fringe metro, medium metro, small metro; and non-metro, defined as micropolitan (10,000 to 49,999 people) and all other non-metropolitan counties), and income-to-poverty ratio defined as ratio of family income to poverty threshold (more than or equal to 2, 0–1.99).

### 2.5. Statistical Analysis

As NHIS is a sample survey of the civilian noninstitutionalized population and not all selected individuals respond, sampling weights were applied to account for the sampling design and nonresponse, ensuring production of nationally representative estimates [24,25]. The weighting process involves several steps: (1) calculation of a base weight, defined as the inverse of the probability of selection, and (2) adjustment of base weights to account for nonresponse patterns across different household and person-level subgroups [24,25]. We pooled the 2019 and 2022 NHIS data and created an adjusted sampling weight for the pooled data. Specifically, the original Sample Adult survey weight (WTFA_A) was divided by the number of survey years pooled (two in our case), resulting in the adjusted weight WTFA_A_adj = WTFA_A/2. This approach ensures that the sum of the pooled weights reflects the average annual U.S. population across the pooled survey years, rather than the cumulative population across both survey years [31]. The adjusted sampling weight was used in all survey-weighted analyses, including PROC SURVEYFREQ for descriptive estimates and PROC SURVEYLOGISTIC for multivariable logistic regression analyses. Complex survey design was accounted for using SAS SURVEY procedures incorporating sampling weights, stratification (PSTRAT), and clustering (PPSU).

Descriptive statistics stratified by HPV vaccination status were reported using the number of participants, weighted percentages, 95% confidence intervals (CIs), and the Rao–Scott Chi-square (*χ*^2^) test for group comparisons. We included relevant covariates in our model [16,17,21]. We conducted survey-weighted bivariable and multivariable logistic regression analyses to examine the association between HPV vaccination and cancer survivorship in the US. In the multivariable logistic regression model, we adjusted for age, sex, race/ethnicity, education, smoking status, housing, US-born status, insurance, location, and income-to-poverty ratio. Because HPV vaccination recommendations and opportunities historically differed by age and sex, we included an interaction term (age*sex) in our multivariable logistic regression analysis. Multicollinearity among covariates was assessed using generalized variance inflation factors (GVIFs) accounting for degrees of freedom. For the multivariable analysis, respondents with “Missing” responses for categorical covariates were not excluded. Instead, these responses were retained as a separate “Missing” category in the regression model. Therefore, all 20,917 participants were included in the multivariable analysis. This approach allowed us to preserve the available sample size. All covariates included in the multivariable model were selected *a priori* based on the study objectives, prior literature, and their potential relevance as sociodemographic and behavioral factors associated with HPV vaccination and cancer history.

All statistical analyses were conducted using SAS version 9.4 (SAS Institute Inc., Cary, NC, USA). Statistical significance was determined using a two-sided *p*-value of <0.05. We adhered to the ‘Strengthening the Reporting of Observational Studies in Epidemiology’ (STROBE) guidelines to ensure ethical and scientific integrity in reporting [32].

## 3. Results

A total of 20,917 age-eligible adults (mean [SD] age (years): 31.41 [13.58]) were included, of whom 489 (2.0%) were cancer survivors of any type. Among these cancer survivors, 19.3% (*n* = 99) had received the HPV vaccination (Table 1). Overall, a higher proportion of individuals included in this study had no personal history of cancer diagnosis of any type (20,423, 98.0%), were aged 27–45 years (16,048, 68.7%), were female (11,194, 50.9%), were non-Hispanic White (12,117, 60.7%), had some college or a bachelor’s education (11,426, 52.1%), had never smoked (14,593, 72.3%), were born in the US (16,708, 81.7%), were insured (17,926, 84.2%), were living in metro areas (18,212, 87.8%), and had an income-poverty ratio of more than two (14,726, 68.9%) (Table 1).

Predominantly, adults who received HPV vaccination were without a personal history of any cancer diagnosis (5317, 27.8%), aged 18–26 years (2336, 47.2%), female (3773, 36.2%), had some college or a bachelor’s education (3304, 31.5%), were never smokers (4240, 31.1%), US-born (4684, 30.2%), insured (4917, 29.7%), living in metro areas (4820, 28.2%), and had an income poverty ratio greater than two (3845, 28.1%) (Table 1).

We performed survey-weighted multivariable logistic regression to estimate the association between a personal history of any cancer and receipt of HPV vaccination, adjusting for age, sex, race/ethnicity, education, smoking status, housing, US-born status, insurance, location, and income-to-poverty ratio. Individuals with a personal history of cancer diagnosis of any type had 33% lower odds of receiving HPV vaccination compared to adults without a personal history of cancer diagnosis (Adjusted Odds Ratio [aOR]: 0.67, 95% CI: 0.52–0.87, *p* = 0.003). Also, individuals living in non-metro areas had 23% lower odds than those living in metro areas (aOR: 0.77, 95% CI: 0.66–0.89, *p* < 0.001). Current smokers had 18% lower odds of HPV vaccination (aOR: 0.82, 95% CI: 0.71–0.94, *p* = 0.005), and former smokers had 25% lower odds of HPV vaccination (aOR: 0.75, 95% CI: 0.67–0.84, *p* < 0.001) than non-smokers. Non-US-born individuals had 46% lower odds of receiving HPV vaccination than US-born individuals (aOR: 0.54, 95% CI: 0.47–0.61, *p* < 0.001). Uninsured individuals had 40% lower odds of receiving HPV vaccination than insured individuals (aOR: 0.60, 95% CI: 0.52–0.69, *p* < 0.001) (Table 2).

Furthermore, individuals who were aged 18–26 years had 449% higher odds (aOR: 5.49, 95% CI: 4.73–6.37, *p* < 0.001) than those aged 27–45 years. Females had 218% higher odds of HPV vaccination than males (aOR: 3.18, 95% CI: 2.84–3.56, *p* < 0.001). Individuals with higher education, including those with some college/bachelor’s education, had 57% higher odds of HPV vaccination (aOR: 1.57, 95% CI: 1.41–1.74, *p* < 0.001), and those with a graduate degree or higher had 91% higher odds of HPV vaccination (aOR: 1.91, 95% CI: 1.65–2.22, *p* < 0.001) than individuals with up to high school education. Individuals living in rented houses had 18% higher odds of receiving HPV vaccination than homeowners (aOR: 1.18, 95% CI: 1.08–1.29, *p* < 0.001). Non-Hispanic others had 40% higher odds of receiving HPV vaccination compared to non-Hispanic Whites (aOR: 1.40, 95% CI: 1.11–1.75, *p* = 0.004). (Table 2).

The age-by-sex interaction was statistically significant; specifically, females aged 18–26 years had 11.5 times higher odds of HPV vaccination compared with males aged 27–45 years (aOR = 11.50, 95% CI: 10.01–13.21, *p* < 0.001; Table 2). No evidence of multicollinearity was observed among the covariates, with standardized GVIF values ranging from 1.00 to 1.34.

## 4. Discussion

In our population-based study using a nationally representative sample, we found that only 19.3% of the cancer survivors had ever received the HPV vaccination in the United States. This low rate of receipt of HPV vaccination among cancer survivors is consistent with findings from previous studies [16,17,21]. Cancer survivors face barriers to HPV vaccination, such as limited knowledge about HPV vaccination, limited discussion with their healthcare professionals or oncologists, concerns about vaccine safety in the context of their cancer history, vaccine hesitancy, and family-based decision-making processes [33,34,35]. Other system-level barriers include limited access to patients’ immunization history, lack of HPV vaccine availability at healthcare professionals’ or oncologists’ healthcare facilities, and operational challenges to vaccine delivery, such as delays in coordinating between pharmacies and clinics [35]. Oncologists are often the preferred source of HPV vaccination recommendations among cancer survivors, underscoring their critical role in promoting HPV vaccination uptake [34]. Primary care professionals found a greater level of comfort in caring for cancer survivors when collaborating with oncologists or cancer centers [36,37]. Therefore, strengthening collaboration between oncologists and primary care professionals may improve HPV vaccine delivery and continuity of preventive care among cancer survivors. To address these multilevel barriers, strategies such as providing clear HPV vaccination recommendations, and the use of digital patient reminder systems have been suggested for increasing HPV vaccination among cancer survivors [38]. Also, establishing clearly defined roles for oncology care teams in vaccine education and delivery, including addressing safety concerns and potential interactions with ongoing cancer treatments, is essential for improving HPV vaccination coverage among cancer survivors.

Consistent with previous studies, our study also found that females were more likely to receive the HPV vaccination than males [16,17,21]. This disparity could be due to the frequent use of healthcare services by women [39], the earlier approval of the HPV vaccination for females (2006) before males (2009) [40,41], and historically female-focused HPV social marketing campaigns [42]. Furthermore, a study among 879 cancer survivors in North Carolina found that female cancer survivors completed the three-dose HPV vaccination series at a higher rate than male cancer survivors [17]. Notably, a large study among 24,363 childhood cancer survivors found that male cancer survivors had a higher risk than female survivors for developing HPV-associated subsequent malignant neoplasms. There is an urgent need for gender-inclusive HPV vaccination programs that target both male and female cancer survivors [43]. We found that individuals aged 18–26 years were more likely to receive HPV vaccination than those aged 27–45 years. In 2019, ACIP introduced shared clinical decision-making for HPV vaccination among adults aged 27–45 years, whereas routine catch-up vaccination remained recommended through age 26 years [27]. Because our study included NHIS data collected in 2019 and 2022, differences in the implementation and uptake of shared clinical decision-making may have contributed to the lower vaccination receipt observed among adults aged 27–45 years. Furthermore, we found individuals living in rented housing were more likely to receive the HPV vaccination than those who owned a house. Renters are more likely to be concentrated in urban areas, where healthcare access is substantially higher than in rural areas, where homeowners are more likely to reside. Although we accounted for urban/rural residence in our analyses, residual confounding may explain this finding and warrants further exploration.

We found that foreign-born individuals were less likely to receive HPV vaccination, consistent with findings from previous studies [44,45,46,47]. This disparity could be attributed to poor access and utilization of healthcare services [48,49]. Additionally, the presence of language barriers or cultural stigmas about sexually transmitted infections may also contribute to lower receipt of HPV vaccination among foreign-born individuals [44]. Furthermore, we found that individuals with higher educational attainment were more likely to receive the HPV vaccination, a finding consistent with existing studies [44,50]. This may be explained by greater awareness of HPV infection and HPV vaccination among individuals with higher educational attainment [51]. In our study, about 37% of participants had attained high school education or less, highlighting the need for targeted public health interventions to increase HPV vaccination among individuals with lower educational attainment. Additionally, we found that uninsured individuals were less likely to receive HPV vaccination, highlighting the critical role of health insurance in facilitating access to HPV vaccination. Previous studies have shown that the uninsured are less likely to initiate HPV vaccination [44,52,53,54,55], Given the importance of insurance coverage for HPV vaccination, our findings further support the potential benefits of the Affordable Care Act (ACA) in expanding insurance coverage and improving access to preventive healthcare services, such as HPV vaccination [56]. Also, we found that individuals who were current or former smokers were less likely to receive the HPV vaccination. This could be due to lower awareness and knowledge about HPV among smokers [57], reduced trust in healthcare professionals [58], and less frequent visits to primary care services [59]. Therefore, proactive strategies to increase HPV vaccination uptake should prioritize improving awareness and access among individuals who are foreign-born, have lower educational attainment, are uninsured, and smoke, using segmented community-centered, culturally and linguistically appropriate approaches tailored to meet the specific needs of each group.

We found that individuals categorized as non-Hispanic others, including non-Hispanic American Indian and Alaska Native (AIAN), single or multiple races, and others, were less likely to have received the HPV vaccination. According to the Cancer Statistics 2026, the AIAN population experiences the highest cancer incidence and mortality rates in the United States [9]. Previous studies have found that the major barriers to HPV vaccination among AIAN populations in the United States were concerns about the safety and efficacy of the HPV vaccination, limited awareness and knowledge about HPV vaccination, lack of healthcare professionals’ recommendations, mistrust of the medical system, and cultural beliefs [60,61,62]. The higher odds of HPV vaccination observed among individuals classified as non-Hispanic others may reflect differences in healthcare utilization, provider recommendation practices, follow-up, or targeted vaccination outreach. However, since the AIAN individuals were combined with other racial/ethnic groups in our analysis, our findings cannot determine whether the observed association specifically reflects HPV vaccination patterns among AIAN individuals.

We found that individuals living in non-metro areas were less likely to receive the HPV vaccination. Consistent with our findings, a study conducted in Minnesota found that rural populations were less likely to utilize preventive healthcare services than their urban counterparts [63]. Additionally, previous studies have shown that rural residents have lower access to health information sources, including healthcare professionals and mass media, less reliable internet access, and lower utilization of health information technologies (HIT), such as online health information management and electronic communication with healthcare professionals, compared to urban residents [64,65]. Furthermore, rural communities often face shortages of healthcare professionals and limited access to healthcare services, which may further hinder access to vaccination services and contribute to disparities in HPV vaccination coverage in rural areas [66,67]. Rural residents have lower awareness and knowledge of HPV, its cancer-causing potential, and HPV vaccination [68]. A scoping review identified facilitators to healthcare access among immigrant populations in rural America, including community-based initiatives, culturally responsive care, and policy-driven supports such as Medicaid and vaccination programs [69]. These findings highlight the need for targeted interventions to address the healthcare needs of minority and rural populations through enhanced healthcare professionals’ engagement, improved preventive healthcare services, and culturally tailored HPV vaccination outreach efforts.

### Strengths and Limitations

This study has several strengths. First, we used a nationally representative sample of the United States’ noninstitutionalized adult population, thereby increasing the generalizability of our findings. Second, the NHIS provides comprehensive information on demographic, socioeconomic, healthcare access, and behavioral characteristics, allowing adjustment for several potential confounding factors. Furthermore, our analytical sample comprised 20,917 individuals, providing substantial statistical power to examine the association between a personal history of cancer and receipt of HPV vaccination. Third, we accounted for the NHIS complex survey design using sampling weights, strata (PSTRAT), and clusters (PPSU). Fourth, we adjusted for sociodemographic, socioeconomic, healthcare, and behavioral characteristics as covariates, including age, sex, race/ethnicity, education, metropolitan status, smoking, U.S. nativity, housing, health insurance, and income-to-poverty ratio. We also evaluated age-by-sex interaction, which is particularly relevant because HPV vaccination recommendations and opportunities historically differed by age and sex.

Study limitations need to be considered before interpreting the findings. First, the cross-sectional nature of the NHIS data limits our ability to establish the temporal sequence between cancer diagnosis and HPV vaccination. Therefore, we cannot draw causal inferences. Second, we utilized self-reported measures that are susceptible to recall, social desirability, and survivorship biases. Since the outcome measured lifetime receipt of at least one HPV vaccine shot, we were unable to distinguish vaccine initiation from completion of the recommended series. Third, the small number of cancer survivors in the analytic sample limited our ability to examine cancer subtypes separately. Therefore, all cancer survivors were grouped into a single category, including HPV-associated and non-HPV-associated cancers; NHIS does not provide sufficient clinical information to determine cancer stage, treatment status, time since diagnosis, or current immune status. Therefore, we could not distinguish active from remote cancer or immunocompromised from non-immunocompromised cancer survivors. This heterogeneity may have influenced HPV vaccination receipt and limits the interpretation of our findings for specific cancer survivor populations.

## 5. Conclusions

In conclusion, we found that HPV vaccination receipt was lower among individuals with a personal history of cancer diagnosis of any type compared to individuals without a history of cancer diagnosis. Future longitudinal studies incorporating detailed clinical information, including cancer diagnosis date, cancer type and treatment history, immune status, and timing of HPV vaccination, are needed to establish the temporal relationship between cancer diagnosis and HPV vaccination. Nonetheless, given the growing number of cancer survivors due to recent advances in early detection and treatment, and the availability of a safe and effective HPV vaccine, strategies to increase HPV vaccination among cancer survivors should be a priority to prevent the risk of developing new HPV-associated cancers.

## Figures and Tables

**Table 1:** Descriptive statistics of age-eligible adults aged 18–45 years, stratified by HPV vaccination status (*n* = 20,917).

Characteristics	Total		Ever Had HPV Vaccine				*p*-Value
	*n* = 20,917		Yes *n* = 5416 (27.6%)		No *n* = 15,501 (72.4%)		
	*n* (wtd%)	95% CI	*n* (wtd%)	95% CI	*n* (wtd%)	95% CI)	
Cancer Survivor							<0.0001
Yes	489 (2.0)	(1.8–2.2)	99 (19.3)	(15.3–23.3)	390 (80.7)	(76.7–84.7)	
No	20,423 (98.0)	(97.8–98.2)	5317 (27.8)	(26.9–28.6)	15,106 (72.2)	(71.4–73.1)	
Age (years)							<0.0001
18–26	4869 (31.3)	(30.5–32.2)	2336 (47.2)	(45.4–49.0)	2533 (52.8)	(51.0–54.6)	
27–45	16,048 (68.7)	(67.8–69.5)	3080 (18.6)	(17.9–19.4)	12,968 (81.4)	(80.6–82.1)	
Sex							<0.0001
Male	9719 (49.1)	(48.3-–49.9)	1642 (18.6)	(17.6–19.6)	8077 (81.4)	(80.4–82.4)	
Female	11,194 (50.9)	(50.1–51.7)	3773 (36.2)	(35.0–37.5)	7421 (63.8)	(62.5–65.0)	
Race/ethnicity							<0.0001
Non-Hispanic White	12,117 (60.7)	(59.1–62.3)	3191 (28.5)	(27.4–29.7)	8926 (71.5)	(70.3–72.6)	
Non-Hispanic Black	2433 (14.1)	(13.1–15.1)	634 (28.2)	(25.8–30.6)	1799 (71.8)	(69.4–74.2)	
Hispanic	2632 (14.4)	(13.1–15.7)	655 (25.4)	(23.4–27.4)	1977 (74.6)	(72.6–76.6)	
Non-Hispanic Asian	1530 (7.0)	(6.4–7.6)	372 (25.3)	(22.6–28.0)	1158 (74.7)	(72.0–77.4)	
Non-Hispanic others *	702 (3.8)	(3.3–4.3)	238 (36.9)	(32.3–41.5)	464 (63.1)	(58.5–67.7)	
Education Level							<0.0001
Up to High School	6424 (36.7)	(35.6–37.8)	1295 (22.3)	(21.0–23.6)	5129 (77.7)	(76.4–79.0)	
Some college/Bachelor	114,26 (52.1)	(51.1–53.1)	3304 (31.5)	(30.3–32.6)	8122 (68.5)	(67.4–69.7)	
Graduate degree or higher	2975 (10.6)	(10.0–11.1)	804 (27.6)	(25.7–29.4)	2171 (72.4)	(70.6–74.3)	
Smoking Status							<0.0001
Current	2710 (12.7)	(12.1–13.3)	495 (19.0)	(17.2–20.8)	2215 (81.0)	(79.2–82.8)	
Former	3325 (15.0)	(14.4–15.6)	618 (18.5)	(16.9–20.0)	2707 (81.5)	(80.0–83.1)	
Never	14,593 (72.3)	(71.5–73.1)	4240 (31.1)	(30.0–32.2)	10,353 (68.9)	(67.8–70.0)	
Housing							<0.0001
Own house	10,609 (57.5)	(56.3–58.6)	2475 (26.5)	(25.4–27.6)	8134 (73.5)	(72.4–74.6)	
Rent house	9246 (42.5)	(41.4–43.7)	2692 (29.3)	(28.0–30.6)	6554 (70.7)	(69.4–72.0)	
Born in US							<0.0001
Yes	16,708 (81.7)	(80.8–82.6)	4684 (30.2)	(29.2–31.1)	12,024 (69.8)	(68.9–70.8)	
No	3696 (18.3)	(17.4–19.2)	603 (16.2)	(14.8–17.7)	3093 (83.8)	(82.3–85.2)	
Insurance							<0.0001
Yes	17,926 (84.2)	(83.3–85.0)	4917 (29.7)	(28.8–30.6)	13,009 (70.3)	(69.4–71.2)	
No	2924 (15.8)	(15.0–16.7)	486 (16.5)	(14.8–18.2)	2438 (83.5)	(81.8–85.2)	
Place of residence							<0.0001
Metro	18,212 (87.8)	(86.8–88.9)	4820 (28.2)	(27.3–29.2)	13,392 (71.8)	(70.8–72.7)	
Non-metro	2705 (12.2)	(11.1–13.2)	596 (22.8)	(20.4–25.2)	2109 (77.2)	(74.8–79.6)	
Income-to-Poverty ratio							0.058
0–1.99	6191 (31.1)	(30.1–32.2)	1571 (26.4)	(24.9–27.9)	4620 (73.6)	(72.1–75.1)	
More than or equal to 2	14,726 (68.9)	(67.8–69.9)	3845 (28.1)	(27.1–29.1)	10,881 (71.9)	(70.9–72.9)	

Missing observations: Seven for cancer survivor, 4 for sex, 1571 for race/ethnicity, 98 for education, 298 for smoking, 1112 for housing, 541 for US-born, 35 for insurance. Unweighted frequencies (*n*) and survey-weighted percentages (wtd%) are presented. *p*-values were calculated using the Rao–Scott Chi-square test. *p*-values are considered significant at 0.05. * Non-Hispanic other includes non-Hispanic American Indian or Alaska Native (AIAN), single or multiple races, and others.

**Table 2:** Multivariable logistic regression analysis of HPV vaccine receipt among age-eligible adults (*n* = 20,907).

Characteristics	Adjusted OR (95% CI)	*p*-Value
Cancer Survivor		
No	Reference	Reference
Yes	0.67 (0.52–0.87)	0.003
Age (years)		
27–45	Reference	Reference
18–26	5.49 (4.73–6.37)	<0.001
Sex		
Male	Reference	Reference
Female	3.18 (2.84–3.56)	<0.001
Age*Sex		
18–26 years Females	Reference	Reference
27–45 years Males	11.50 (10.01–13.21)	<0.001
Race/ethnicity		
Non-Hispanic White	Reference	Reference
Non-Hispanic Black	0.95 (0.82–1.09)	0.436
Hispanic	0.98 (0.86–1.11)	0.735
Non-Hispanic Asian	1.00 (0.84–1.20)	0.961
Non-Hispanic others *	1.40 (1.11–1.75)	0.004
Education		
Up to High School	Reference	Reference
Some college/Bachelor	1.57 (1.41–1.74)	<0.001
Graduate degree or higher	1.91 (1.65–2.22)	<0.001
Smoking Status		
Never	Reference	Reference
Current	0.82 (0.71–0.94)	0.005
Former	0.75 (0.67–0.84)	<0.001
Housing		
Own house	Reference	Reference
Rent house	1.18 (1.08–1.29)	<0.001
US-Born		
Yes	Reference	Reference
No	0.54 (0.47–0.61)	<0.001
Insurance		
Yes	Reference	Reference
No	0.60 (0.52–0.69)	<0.001
Place of residence		
Metro	Reference	Reference
Non-metro	0.77 (0.66–0.89)	<0.001
Income-to-Poverty ratio		
More than or equal to 2	Reference	Reference
0–1.99	0.96 (0.86–1.07)	0.475

Missing observations: Seven for cancer survivor, 4 for sex, 1571 for race/ethnicity, 98 for education, 298 for smoking, 1112 for housing, 541 for US-born, 35 for insurance. *p*-values are two-sided and considered significant at 0.05. * Non-Hispanic Others included non-Hispanic American Indian or Alaska Native (AIAN), single or multiple races, and others.

## Data Availability

Publicly available National Health Interview Survey (NHIS) Sample Adult Interview datasets analyzed in this study can be found at: 1. 2019 NHIS: https://www.cdc.gov/nchs/nhis/documentation/2019-nhis.html. 2. 2022 NHIS: https://www.cdc.gov/nchs/nhis/documentation/2022-nhis.html.

## Abbreviations

The following abbreviations are used in this manuscript:

CDC	Centers for Disease Control and Prevention
ACIP	Advisory Committee on Immunization Practices
FDA	Food and Drug Administration
NCHS	National Center for Health Statistics
NHIS	National Health Interview Survey
HPV	Human Papillomavirus
ACA	Affordable Care Act
CI	Confidence Interval
